# Sarcopenia trajectories and associated factors after lung transplantation: a growth mixture model study

**DOI:** 10.3389/fmed.2026.1920710

**Published:** 2026-09-01

**Authors:** Mengshan Xie, Fei Zeng, Fangfang Hao, Peipei Gu, Jiangshuyuan Liang, Yandie Wang

**Affiliations:** Department of Nursing, The Second Affiliated Hospital of Zhejiang University School of Medicine, Hangzhou, China

**Keywords:** growth mixture model, hand strength, lung transplantation, risk factors, sarcopenia, trajectory

## Abstract

**Background:**

Sarcopenia is common after lung transplantation and is associated with poor outcomes, yet its postoperative course is dynamic and may differ between recipients. We aimed to identify latent classes of sarcopenia trajectories during the first year after lung transplantation and the factors associated with them.

**Methods:**

In this retrospective cohort study, 264 recipients who underwent lung transplantation at a tertiary hospital between June 2023 and March 2025 were assessed with the Ishii score at up to six time points (preoperatively and at 1 week and 1, 3, 6, and 12 months). Growth mixture modeling identified latent trajectory classes, missing data were handled with full information maximum likelihood, and multinomial logistic regression identified associated factors.

**Results:**

The Ishii score followed an inverted V-shaped course, with the prevalence of probable sarcopenia rising from 55.7% preoperatively to 95.6% at 1 week and declining to 28.4% at 12 months. Three Ishii score trajectories were identified: low-risk (40.5%), moderate-risk (36.7%), and high-risk (22.7%). Older age (OR 1.08), lower preoperative BMI (OR 0.81), lower preoperative FEV1%pred (OR 0.97), longer ICU stay (OR 1.11), and a higher NRS 2002 score (OR 1.39) were independently associated with the high-risk trajectory (all *p* < 0.05). Recipients in the high-risk trajectory also had the poorest physical function at one year.

**Conclusion:**

Postoperative Ishii score trajectories were markedly heterogeneous among lung transplant recipients. Repeated Ishii screening with trajectory-based, individualized management may support the early identification and targeted care of high-risk recipients.

## Introduction

Lung transplantation is an established therapeutic option for end-stage lung disease. The primary indications include interstitial lung disease and chronic obstructive pulmonary disease (1). With 1-year survival now reaching 88.4% (2), attention has turned to recipients’ longer-term functional recovery. Sarcopenia is a progressive skeletal muscle disorder characterized primarily by low muscle strength, with reduced muscle quantity confirming the diagnosis and low physical performance indicating severity (3). It is associated with falls, impaired activities of daily living, loss of functional independence, and increased healthcare costs (4). In lung transplant recipients, sarcopenia is associated with increased all-cause mortality (hazard ratio 5.8), poorer lung function (5), and greater perioperative morbidity (6). Together, these associations identify sarcopenia as a marker of poor post-transplant outcomes.

Even before transplantation, sarcopenia affects nearly half of candidates with end-stage lung disease, leaving them with little muscle reserve at surgery (7, 8). After transplantation, recipients move from intensive care through the general ward to home, and over this period surgical stress, immobilization, and corticosteroid therapy contribute to the loss of skeletal muscle (9). Across the first postoperative year, sarcopenia follows a dynamic course rather than a steady decline or recovery. Muscle mass tends to improve only gradually, and the extent of recovery differs markedly between recipients. Nearly half remain sarcopenic at 12 months, and some take years to recover (10). Yet sarcopenia is modifiable, as exercise and protein supplementation can improve muscle mass, strength, and function (11).

Although sarcopenia after lung transplantation has been increasingly studied, most reports have been cross-sectional, capturing only the group-average status at a single time point. By contrast, longitudinal trajectory analyses have identified four sarcopenia trajectories in community-dwelling older adults (12) and two frailty trajectories in lung transplant recipients (13). Sarcopenia and frailty are related but not interchangeable, the former being a disorder specific to skeletal muscle that has been regarded as the biological substrate of physical frailty, and the latter a cumulative decline across multiple physiological systems (4). Longitudinal studies in lung transplant recipients have documented an early decline and subsequent recovery in muscle strength (9, 14), as well as gradual improvements in muscle mass, physical performance, and sarcopenia status over several years (10). Taken together, these studies describe the overall course of muscle recovery but do not show whether lung transplant recipients follow distinct trajectories of sarcopenia risk or which factors distinguish them. Whereas muscle strength and physical performance can be measured at the bedside, confirming low muscle mass relies on imaging ill-suited to repeated use. This limitation prompted the development of the Ishii score from age, grip strength, and calf circumference (15). Compared with the insensitive SARC-F questionnaire (3), it is objective and identifies probable sarcopenia with good discrimination in chronic lung disease (16). As a bedside measure, it therefore supports the repeated assessment needed to characterize these trajectories.

Given the heterogeneity of post-transplant sarcopenia and the absence of trajectory data, this study had two aims. The first was to identify latent classes of sarcopenia trajectories from before transplantation to one year afterward using growth mixture modeling. The second was to examine the factors associated with class membership, to inform the early identification of high-risk recipients and the design of trajectory-based interventions.

## Methods

### Study design and participants

We conducted a retrospective cohort study at a tertiary hospital in Zhejiang Province, China. We included all consecutive recipients aged 18 years or older who had undergone single or double lung transplantation between June 2023 and March 2025 and who had grip strength and calf circumference measured at two or more time points from before transplantation to one year afterward, from which the Ishii score was calculated. We excluded recipients with (i) cognitive impairment that precluded assessment; (ii) conditions that interfered with grip strength or calf circumference measurement, such as limb amputation or neuromuscular disease; (iii) conditions that could independently affect skeletal muscle metabolism, such as active malignancy or severe hepatic or renal insufficiency; and (iv) re-transplantation. For growth mixture modeling, a sample of at least 200 is recommended (17), and the final sample of 264 recipients exceeded this threshold. The study was approved by the hospital ethics committee (approval number 2026-0370), which waived the requirement for written informed consent given the retrospective design.

### Sarcopenia assessment

Sarcopenia risk was assessed with the Ishii score, an objective screening tool developed by Ishii et al. (15) that combines age, grip strength, and calf circumference. It is described as a sarcopenia screening method in the revised European consensus (3) and is recommended by the Chinese sarcopenia guideline (18). Grip strength was measured on the dominant side with an electronic hand dynamometer (CAMRY, China), with the participant seated and the elbow flexed at 90°. The maximum of three measurements, taken at 30-s intervals, was recorded in kilograms. Calf circumference was measured with an inelastic tape in a seated position with the knee flexed at 90°, and the larger of the left and right values was recorded in centimeters. Each component was scored according to sex-specific charts and summed, with cutoffs of 105 for men and 120 for women (15, 18). An Ishii score above the sex-specific cutoff was considered a positive screen for probable sarcopenia. In patients with chronic lung disease, the Ishii score at these cutoffs identifies probable sarcopenia with a sensitivity of 71.6%, a specificity of 90.5%, and an area under the receiver operating characteristic curve of 0.81 (16).

### Covariates

Candidate factors were selected on the basis of prior literature and clinical relevance. They were collected with a structured data form covering three domains. Demographic data comprised sex, age, educational level, body mass index (BMI) calculated from preoperative weight and height, and smoking history. Disease and perioperative data comprised the primary diagnosis, transplant type (single or double lung), use of extracorporeal membrane oxygenation (ECMO), diabetes mellitus, hypertension, length of intensive care unit (ICU) stay, postoperative length of stay, and preoperative percent-predicted forced expiratory volume in one second (FEV1%pred). Nutritional and laboratory data comprised the Nutritional Risk Screening 2002 (NRS 2002) score (19), with a score of 3 or more indicating nutritional risk, and preoperative serum albumin. Although age is a component of the Ishii score, it was retained as a candidate covariate because it is a well-recognized risk factor for sarcopenia and poorer post-transplant recovery and is central to clinical risk stratification. Variable categories are presented in Table 1.

**Table 1:** Univariate analysis of demographic and clinical characteristics of lung transplant recipients by sarcopenia trajectory class (*n* = 264).

Variable	Total (*n* = 264)	High-risk (*n* = 60)	Moderate-risk (*n* = 97)	Low-risk (*n* = 107)	*p*
Demographic characteristics					
Male, *n* (%)	227 (86.0%)	49 (81.7%)	84 (86.6%)	94 (87.9%)	0.531
Age (years)	59.0 (52.0, 65.0)	62.0 (57.0, 67.0)	59.0 (52.0, 65.0)	57.0 (50.5, 61.5)	0.002
Educational level, *n* (%)					0.680
Junior high or below	85 (32.2%)	21 (35.0%)	29 (29.9%)	35 (32.7%)	
Senior high/technical	98 (37.1%)	26 (43.3%)	37 (38.1%)	35 (32.7%)	
Junior college	55 (20.8%)	8 (13.3%)	21 (21.6%)	26 (24.3%)	
Bachelor or above	26 (9.8%)	5 (8.3%)	10 (10.3%)	11 (10.3%)	
Smoking history, *n* (%)	71 (26.9%)	18 (30.0%)	31 (32.0%)	22 (20.6%)	0.154
Preoperative clinical characteristics					
Preoperative BMI (kg/m^2^)	20.7 ± 4.0	18.8 ± 4.0	20.7 ± 3.9	21.8 ± 3.7	<0.001
Primary diagnosis, *n* (%)					<0.001
Interstitial lung disease	133 (50.4%)	21 (35.0%)	52 (53.6%)	60 (56.1%)	
COPD	63 (23.9%)	27 (45.0%)	18 (18.6%)	18 (16.8%)	
Pneumoconiosis	36 (13.6%)	3 (5.0%)	14 (14.4%)	19 (17.8%)	
Other	32 (12.1%)	9 (15.0%)	13 (13.4%)	10 (9.3%)	
Diabetes mellitus, *n* (%)	65 (24.6%)	20 (33.3%)	26 (26.8%)	19 (17.8%)	0.067
Hypertension, *n* (%)	39 (14.8%)	13 (21.7%)	15 (15.5%)	11 (10.3%)	0.134
Preoperative FEV1%pred	45.0 (27.9, 58.1)	34.8 (21.4, 46.6)	46.4 (27.7, 58.7)	49.0 (36.8, 59.6)	0.001
NRS 2002 (score)	2.0 (1.0, 4.0)	4.0 (2.0, 5.0)	3.0 (2.0, 4.0)	2.0 (1.0, 3.0)	<0.001
Albumin (g/L)	35.4 (32.5, 38.2)	35.8 (33.3, 38.4)	35.4 (32.5, 37.9)	35.2 (32.2, 38.8)	0.837
Perioperative characteristics					
Double-lung transplantation, *n* (%)	159 (60.2%)	38 (63.3%)	60 (61.9%)	61 (57.0%)	0.666
ECMO, *n* (%)	224 (84.8%)	49 (81.7%)	82 (84.5%)	93 (86.9%)	0.658
ICU stay (d)	4.0 (3.0, 7.0)	5.0 (3.0, 10.5)	5.0 (3.0, 7.0)	4.0 (3.0, 6.0)	0.012
Postoperative stay (d)	31.0 (23.0, 41.0)	34.0 (29.0, 48.8)	30.5 (23.0, 41.0)	29.0 (22.0, 34.5)	0.002

Continuous variables are presented as mean ± SD (normally distributed) or median (P25, P75) (non-normally distributed); categorical variables as *n* (%). *p* values were derived from one-way ANOVA, the Kruskal-Wallis H test, or the chi-square test, as appropriate. BMI, body mass index; ICU, intensive care unit; ECMO, extracorporeal membrane oxygenation; NRS 2002, Nutritional Risk Screening 2002; FEV1%pred, percent-predicted forced expiratory volume in one second. Other comprised bronchiectasis (*n* = 12), bronchiolitis obliterans (*n* = 11), pulmonary arterial hypertension (*n* = 3), and other diagnoses (*n* = 6).

### Data collection and quality control

Data were collected retrospectively from the hospital electronic medical record system. During hospital stays, grip strength and calf circumference were measured by trained ward nurses as part of routine care, following a standardized protocol. Each measurement was assigned to one of six predefined time points relative to transplantation (preoperative [T1], 1 week [T2], 1 month [T3], 3 months [T4], 6 months [T5], and 12 months [T6]). The Ishii score was then calculated from the corresponding grip strength, calf circumference, and age. At T2, some recipients were still in the ICU or too ill to complete grip strength measurement, so their Ishii score was missing for that time point. Because fluid retention is common after lung transplantation, the ward nurses also graded lower-limb edema (0 to 3) at each calf circumference measurement. Time points with grade 2 or higher were flagged. Physical performance at 12 months was assessed with the Short Physical Performance Battery (SPPB). The SPPB was used in binary (SPPB ≤9 indicates functional limitation) and continuous (range, 0–12; lower scores indicate poorer physical performance) forms (18). Two trained researchers extracted the data independently with a predefined form and cross-checked their entries. Discrepancies were resolved by tracing the original records. A third researcher randomly verified 10% of the data and rechecked the accuracy of score calculation.

### Statistical analysis

Growth mixture models were fitted to the Ishii scores in Mplus 8.3 with a random intercept and a random slope. Time was coded in months (0, 0.25, 1, 3, 6, and 12). Linear, quadratic, and cubic functions were compared based on model fit and theoretical plausibility. Models with one to five classes were estimated and compared with the Akaike information criterion (AIC), Bayesian information criterion (BIC), sample-size-adjusted BIC (aBIC), entropy, the Lo–Mendell–Rubin adjusted likelihood ratio test (LMR), and the bootstrapped likelihood ratio test (BLRT). The LMR and BLRT compared the k-class model with the (k–1)-class model. The final model was selected based on the model-fit indices and clinical interpretability, and each class was required to comprise at least 5% of the sample. Lower AIC, BIC, and aBIC values indicated better model fit, whereas higher entropy indicated better classification quality. An average posterior probability of at least 0.70 was required for each class. Missing Ishii scores were handled with full information maximum likelihood, which estimates the model from all available assessments rather than excluding recipients with incomplete data or imputing the missing scores. To assess the plausibility of the missing-at-random assumption, loss of the 12-month assessment was examined in relation to baseline and perioperative characteristics with logistic regression (Supplementary Table S1). Each recipient was then assigned to the trajectory class for which the posterior probability was highest.

Further analyses were performed in R 4.3.2. In the univariate analysis, the recipients’ characteristics were compared across the three trajectory classes: normally distributed continuous variables were expressed as mean ± standard deviation and compared with one-way analysis of variance, non-normally distributed variables as median (P25, P75) and compared with the Kruskal-Wallis H test, and categorical variables as number (percentage) and compared with the χ^2^ test or the Fisher exact test (Table 1). To identify factors independently associated with class membership, a multinomial logistic regression was fitted with the low-risk trajectory as the reference. Factors that differed across the classes in the univariate analysis (*p* < 0.05) were entered. Multicollinearity among these factors was assessed with the variance inflation factor (VIF). All values were below 5, indicating no significant multicollinearity, so no factor was removed. Missing covariate values were handled with multiple imputation (20 imputed datasets pooled using Rubin’s rules). For the exploratory outcome analysis, SPPB scores and the prevalence of functional limitation were compared across the three trajectories using the Kruskal-Wallis H test and the χ^2^ test, respectively (Supplementary Table S4). A two-sided *p* < 0.05 was considered statistically significant.

Five sensitivity analyses were performed. Three applied model-based corrections and examined the stability of the trajectory classes. To correct the distortion of calf circumference by postoperative edema (20), each measurement was adjusted for the inflation attributable to its edema grade, estimated with a linear mixed model of calf circumference on edema grade and time point with a random intercept for recipient. The Ishii score was then recalculated from the corrected values and the growth mixture model refitted. To assess whether the time-invariant age component influenced trajectory classification, the same three-class growth mixture model was refitted using an age-component-removed score calculated by summing the outputs of the sex-specific grip-strength and calf-circumference scoring functions implemented in this study, with neither function re-estimated or rescaled. To address differential loss to follow-up, stabilized inverse-probability weights were derived from a logistic model predicting loss of the 12-month assessment from age at transplantation, sex, ICU stay, NRS 2002 score, and preoperative Ishii score. The growth mixture model was then refitted with these weights among recipients who completed that assessment (Supplementary Table S3). Two further analyses re-estimated the associated factors in restricted subsets. The first excluded recipients with grade 2 or higher edema at any time point. The second was limited to recipients with complete Ishii scores at all six time points (Supplementary Table S5). The three model-based sensitivity analyses were compared with the primary full-information maximum likelihood model, and the two restricted-subset analyses with the primary multinomial logistic regression.

## Results

### Baseline characteristics of participants

In total, 285 consecutive patients underwent lung transplantation at the center during the study period. Of these, 11 were excluded for cognitive impairment, a condition affecting muscle measurement or metabolism, or re-transplantation, and a further 10 had fewer than two Ishii assessments. The remaining 264 recipients formed the analytic cohort, of whom 205 (77.7%) were assessed at all six time points and 59 at two to five. The median age was 59.0 years (52.0, 65.0), and 227 (86.0%) were male. The most common primary diagnosis was interstitial lung disease (*n* = 133, 50.4%), followed by chronic obstructive pulmonary disease (*n* = 63, 23.9%). Overall, 159 recipients (60.2%) received a double lung transplantation and 105 (39.8%) a single lung transplantation. Intraoperative ECMO was used in 224 recipients (84.8%). At baseline, 147 recipients (55.7%) screened positive for probable sarcopenia by the Ishii score. Recipient characteristics are shown in Table 1.

### Heterogeneous sarcopenia trajectories

Among the linear, quadratic, and cubic functions, the cubic function provided the best statistical fit and most closely reflected the expected postoperative pattern of an early increase, subsequent decline, and later slowing of change. Models with one to five classes were then fitted using the cubic function (Table 2). The AIC, BIC, and aBIC reached their lowest values at the five-class model, and the BLRT was significant at every step. The BIC fell by 155 from two to three classes but by only 5 from three to four classes, and entropy declined from 0.906 to 0.881. Given these mixed findings, the model-fit indices, minimum class size, and clinical interpretability were considered together. The LMR test was significant for the three-class model but not for the four-class (*p* = 0.554) or five-class (*p* = 0.200) model, suggesting that the model with one fewer class was preferred in the latter comparisons. The five-class model was additionally excluded because its smallest class comprised only 3.7% of the sample, below the prespecified 5%. The three-class model was therefore selected. Its entropy of 0.906 and average posterior probabilities above 0.94 indicated strong classification precision.

**Table 2:** Model fit indices for the growth mixture models.

Classes	AIC	BIC	aBIC	Entropy	LMR P	BLRT P	Class proportion (%)
1	11907	11953	11912	—	—	—	100.0
2	11761	11826	11769	0.919	<0.001	<0.001	22.8/77.2
3	11589	11671	11598	0.906	<0.001	<0.001	22.3/37.1/40.6
4	11566	11666	11577	0.881	0.554	<0.001	7.7/22.2/30.0/40.2
5	11500	11618	11513	0.887	0.200	<0.001	3.7/9.4/22.5/28.0/36.4

Class proportions are model-estimated and may differ slightly from the modal-assignment counts in Table 1. AIC, Akaike information criterion; BIC, Bayesian information criterion; aBIC, sample-size-adjusted BIC; LMR, Lo–Mendell–Rubin adjusted likelihood ratio test; BLRT, bootstrapped likelihood ratio test.

**Figure 1:**
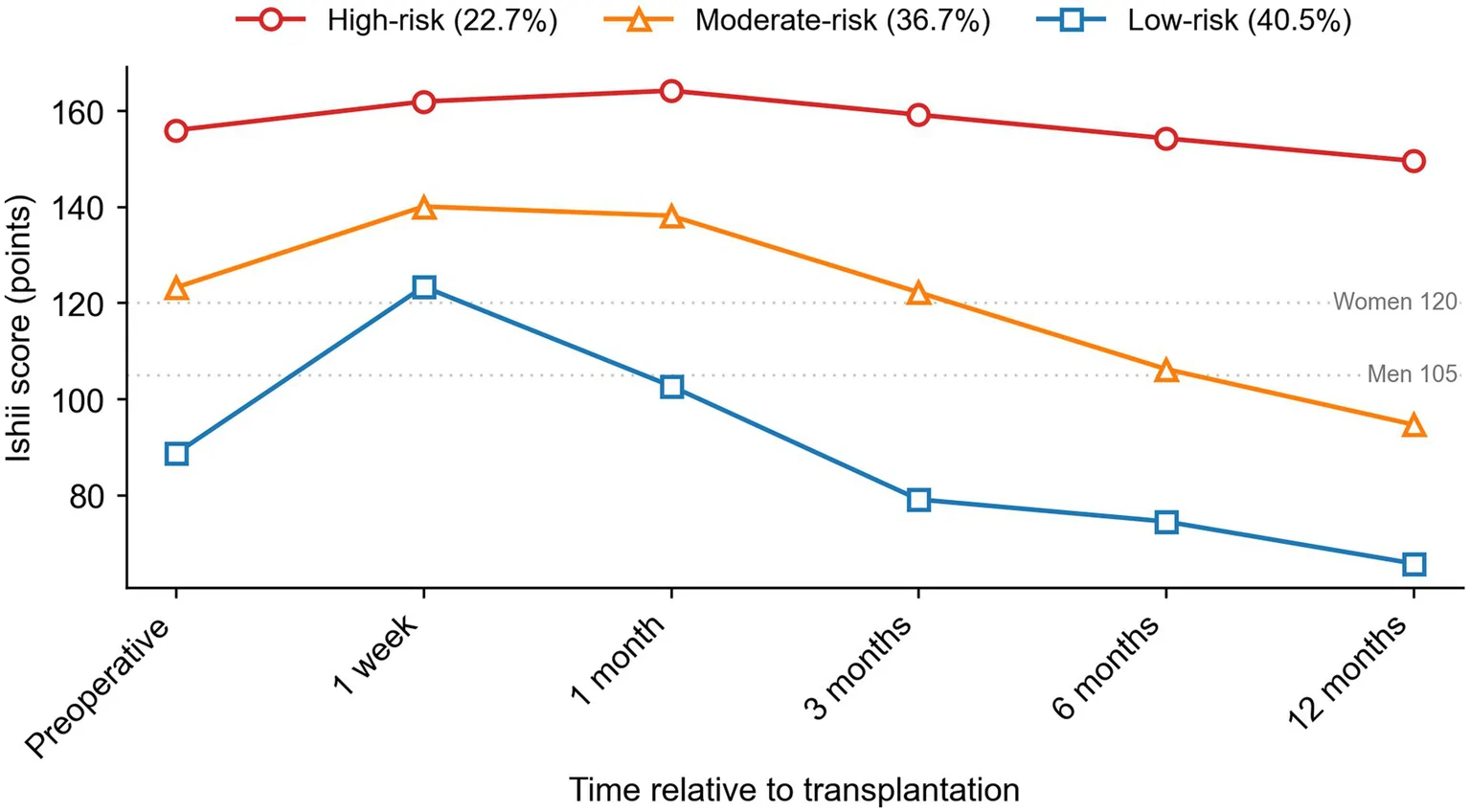
Sarcopenia trajectories of the three latent classes identified by growth mixture modeling. There were 60 recipients in the high-risk trajectory, 97 in the moderate-risk trajectory, and 107 in the low-risk trajectory. Points are class mean Ishii scores at each time point. The dotted horizontal lines mark the Ishii cutoffs for probable sarcopenia (105 for men, 120 for women).

According to their overall Ishii score levels, the three latent classes were labeled the low-risk, moderate-risk, and high-risk trajectories (Figure 1; Supplementary Table S2). The overall Ishii score followed an inverted V-shaped pattern. The prevalence of probable sarcopenia rose from 55.7% (147/264) preoperatively to 95.6% (240/251) at 1 week and then declined to 28.4% (64/225) at 12 months. The high-risk trajectory (*n* = 60, 22.7%) was characterized by high preoperative scores that rose further to a peak between 1 week and 1 month and then declined slowly while remaining above the cutoff throughout. The moderate-risk trajectory (*n* = 97, 36.7%) was characterized by intermediate preoperative scores that rose at 1 week, declined steadily from 1 month onward, and ended near the cutoff by one year. The low-risk trajectory (*n* = 107, 40.5%) was characterized by the lowest preoperative scores, which rose transiently at 1 week, fell rapidly, and remained well below the cutoff thereafter. Similar class proportions were obtained in three further growth mixture models that adjusted calf circumference for edema, used an age-component-removed score, or weighted for differential loss to follow-up (Supplementary Table S3). In the edema-adjusted model, 98.9% of recipients retained their original class assignment, and 94.7% did so in the age-component-removal sensitivity analysis.

### Factors associated with sarcopenia trajectories

In the univariate analysis, several demographic, clinical, and perioperative characteristics differed significantly across the three trajectories. Recipients in the high-risk trajectory were older than those in the other two trajectories. They also entered transplantation with poorer nutritional status, indicated by a lower BMI and a higher NRS 2002 score. Their preoperative lung function was poorer, with a lower FEV1%pred. Chronic obstructive pulmonary disease was more common among recipients in the high-risk trajectory, whereas interstitial lung disease predominated among those in the moderate-risk and low-risk trajectories. After transplantation, recipients in the high-risk trajectory had longer intensive care unit and postoperative stays. Sex, smoking history, comorbidities, transplant type, and ECMO use did not differ significantly among the trajectories (Table 1). In the multivariable analysis, older age was associated with both the high-risk (odds ratio [OR] 1.084, 95% CI 1.036–1.134) and moderate-risk (OR 1.042, 95% CI 1.010–1.076) trajectories, and a higher NRS 2002 score was also associated with both the high-risk (OR 1.392, 95% CI 1.055–1.836) and moderate-risk (OR 1.298, 95% CI 1.046–1.611) trajectories. In contrast, a lower preoperative BMI was associated with the high-risk trajectory (OR 0.810, 95% CI 0.705–0.931), as were a lower preoperative FEV1%pred (OR 0.974, 95% CI 0.948–1.000) and a longer ICU stay (OR 1.109, 95% CI 1.026–1.198) (Table 3). While older age and higher nutritional risk were associated with both adverse trajectories, lower BMI, lower FEV1%pred, and longer ICU stay specifically distinguished the high-risk trajectory. Across both restricted-subset analyses, the parameter estimates were similar to those of the primary analysis, and no additional predictors were identified (Supplementary Table S5).

**Table 3:** Multinomial logistic regression of sarcopenia trajectory classes (*n* = 264).

Variable	High-risk vs low-risk		Moderate-risk vs low-risk	
	OR (95% CI)	*p*	OR (95% CI)	*p*
Age (years)	1.084 (1.036–1.134)	**<0.001**	1.042 (1.010–1.076)	**0.011**
Preoperative BMI	0.810 (0.705–0.931)	**0.003**	0.928 (0.845–1.019)	0.119
Primary diagnosis				
Interstitial lung disease	–		–	
COPD	1.141 (0.382–3.412)	0.814	0.650 (0.260–1.621)	0.355
Other	0.596 (0.180–1.973)	0.397	0.879 (0.391–1.977)	0.755
Preoperative FEV1%pred	0.974 (0.948–1.000)	**0.049**	0.988 (0.970–1.007)	0.215
NRS 2002 score	1.392 (1.055–1.836)	**0.019**	1.298 (1.046–1.611)	**0.018**
ICU stay	1.109 (1.026–1.198)	**0.009**	1.070 (0.994–1.151)	0.074
Postoperative stay	1.020 (0.993–1.047)	0.155	1.005 (0.982–1.029)	0.651

The low-risk trajectory is the reference category. For primary diagnosis, interstitial lung disease is the reference (−) and pneumoconiosis was grouped with “Other.” Statistically significant *p* values are shown in bold. The 95% CI upper bound for FEV1%pred rounds to 1.000 but remains below 1 (*p* = 0.049). OR, odds ratio; CI, confidence interval; BMI, body mass index; FEV1%pred, percent-predicted forced expiratory volume in one second; ICU, intensive care unit; NRS 2002, Nutritional Risk Screening 2002.

### Physical performance across trajectories

In an exploratory analysis, physical performance at 12 months was compared across the three trajectories (Supplementary Table S4). The SPPB score rose from a median of 8 (6, 10) in the high-risk trajectory to 10 (8, 12) in the moderate-risk trajectory and 11 (9, 12) in the low-risk trajectory (*p* < 0.001). Functional limitation, defined as an SPPB score of 9 or below, showed the same gradient, falling from 71.7% (33/46) in the high-risk trajectory to 47.6% (40/84) in the moderate-risk trajectory and 28.4% (27/95) in the low-risk trajectory (*p* < 0.001).

## Discussion

This study used growth mixture modeling to identify three sarcopenia trajectories during the first year after lung transplantation: low-risk, moderate-risk, and high-risk. Factors associated with the high-risk trajectory included older age, lower preoperative BMI and FEV1%pred, longer ICU stay, and a higher NRS 2002 score.

In this cohort, the overall Ishii score followed an inverted V-shaped course, deteriorating sharply within the first week after transplantation and then improving gradually over the first year, indicating that Ishii-defined sarcopenia risk changed dynamically rather than remaining fixed. The marked early deterioration probably reflects the combined burden of limited preoperative muscle reserve, surgical trauma, perioperative immobilization, and immunosuppression. A comparable course has been documented after lung transplantation, in which quadriceps strength falls to a nadir within the first postoperative weeks and then recovers over the following year (14), and muscle force declines acutely after surgery but is partly reversed by exercise training (9). Nikkuni et al. (10) likewise reported that some recipients with post-transplant sarcopenia regain physical function over time. This average course, however, conceals substantial heterogeneity between recipients, which growth mixture modeling resolved into three distinct trajectories.

Recipients were classified into low-risk (40.5%), moderate-risk (36.7%), and high-risk (22.7%) trajectories. They varied in both level and shape, confirming substantial heterogeneity in the postoperative course of sarcopenia. This heterogeneity aligns with the distinct frailty trajectories described in lung transplant recipients (13). These divergent courses likely reflect differences in baseline reserve and in the capacity for muscle regeneration. Recipients in the low-risk trajectory entered transplantation with adequate reserve. Once the acute stress resolved, muscle protein synthesis could resume, in keeping with the fall of their scores well below the cutoff within a year. Among recipients in the moderate-risk trajectory, reserve appeared to be consumed more rapidly, and persistent low-grade inflammation may have impaired full regeneration (21), in keeping with the gradual decline of their scores toward the cutoff. Recipients in the high-risk trajectory entered transplantation with a poorer baseline and had longer ICU and postoperative stays. In this setting, glucocorticoid exposure may further accelerate muscle protein breakdown (22) and rising intramuscular fat infiltration may impair regeneration (23), mechanisms consistent with their persistently elevated scores and the limited reversibility of the deficit.

Several baseline and perioperative factors distinguished the trajectories. Older age was associated with both the high-risk (OR 1.084) and the moderate-risk (OR 1.042) trajectories, consistent with the well-recognized decline in muscle mass and strength with advancing age (4). This decline partly reflects anabolic resistance, a state in which the protein-synthetic response to amino acids and exercise is blunted by impaired mTOR (TORC1) signalling (24). In parallel, the accumulation of senescent cells sustains a chronic low-grade inflammation known as inflammaging, which activates NF-κB and upregulates muscle ring finger 1 (MuRF1), a key factor promoting muscle protein degradation (21, 24). These age-related changes are particularly relevant to lung transplant recipients, who are frequently middle-aged or older (2). Entering transplantation with diminished anabolic reserve, such recipients may recover muscle less readily, consistent with the observed association between older age and membership in a higher-risk trajectory.

Poorer nutritional status distinguished the high-risk trajectory, indexed by a lower BMI (OR 0.810) and a higher NRS 2002 score (OR 1.392), with the latter also separating the moderate-risk trajectory (OR 1.298). The two indices reflect different aspects of nutrition, BMI through weight status and the NRS 2002 through dynamic risk such as recent weight loss and the severity of illness. Because BMI is one component of the NRS 2002 score, their independent associations suggest that nutrition is linked to the trajectories through more than one pathway. In advanced lung disease, the increased work of breathing raises resting energy expenditure while intake fails to keep pace, producing a negative energy balance that erodes fat-free mass (25). Malnutrition is correspondingly common in transplant candidates, affecting nearly half and presenting most often as reduced muscle mass (26). Entering transplantation with little reserve, these recipients face surgical stress and immunosuppression that further suppress muscle protein synthesis and accelerate atrophy (4). Rebuilding muscle draws on reserves they have already spent, so the deficit may persist rather than recover, in line with their high-risk trajectory. Yet neither index measures muscle directly, so a normal BMI or NRS 2002 score can mask depleted muscle. Computed tomography illustrates this gap, as recipients with low muscle but high fat stayed far longer in the ICU than those with high muscle and low fat (25 vs. 3.5 days) (6). Unlike age, nutrition is modifiable, and screening with both BMI and the NRS 2002 offers a practical way to flag recipients who would benefit from earlier nutritional support (11).

Worse preoperative lung function marked the high-risk trajectory (OR 0.974). Consistent with this, sarcopenic patients with idiopathic pulmonary fibrosis show a lower FEV1 (27). However, Kanezaki et al. (28) found no such difference in interstitial lung disease, probably reflecting a narrower and less severe range of lung function than in transplant candidates. Among transplant candidates, greater disease severity imposes a heavier burden of chronic hypoxia, which impairs skeletal muscle mitochondria and oxidative capacity (29), while dyspnea and reduced exercise tolerance curtail physical activity and promote disuse atrophy (30). These effects accumulate over the long course of the disease, so recipients with the lowest FEV1%pred reach transplantation with the largest accumulated muscle deficit, consistent with the observed association between poorer lung function and high-risk trajectory membership. Sarcopenia in lung disease involves the respiratory muscles as well (28), so adding inspiratory muscle training to rehabilitation may particularly benefit these recipients.

After surgery, a longer ICU stay was associated with higher odds of the high-risk trajectory (OR 1.109), which may reflect both a more severe overall course and the muscle loss that accompanies prolonged critical illness. Consistent with this, muscle force after lung transplantation is directly related to time spent in the ICU (9). Prolonged immobilization deprives skeletal muscle of mechanical loading, its main anabolic stimulus, and may progress to ICU-acquired weakness, which encompasses critical illness polyneuropathy and critical illness myopathy (31). The acute inflammation and negative nitrogen balance of critical illness drive proteolysis, with as much as 10% of muscle mass lost in the first week (32). Such a long ICU stay also signals a more complicated course and a longer exposure to this catabolic state, which may make the deficit harder to reverse and is consistent with the high-risk trajectory. Although the benefit of early active mobilization remains uncertain (33), passive neuromuscular electrical stimulation may help limit loss in recipients who cannot yet participate.

Physical function at one year was also poorest among recipients in the high-risk trajectory. Taken together, these findings indicate that sarcopenia after lung transplantation should be managed according to trajectory rather than as a uniform condition. Repeated Ishii screening across the first postoperative year can identify which trajectory a recipient is following and flag those who fail to recover, while the associated factors help target the recipients at highest risk for earlier and more intensive support. Because no single index reliably captures muscle status, adding direct measures of muscle mass and function would improve detection. Much of this risk is already present before surgery, so muscle protection could begin before transplantation with prehabilitation and continue through critical illness, pairing nutritional support with exercise throughout. As part of routine post-transplant rehabilitation, these elements could form a closed loop of dynamic screening, risk stratification, graded intervention, and continued monitoring across the first postoperative year.

### Strengths and limitations

To our knowledge, this study is the first to apply growth mixture modeling to sarcopenia after lung transplantation, and it identifies distinct trajectory classes that cross-sectional designs cannot reveal. We used full information maximum likelihood to retain the whole sample rather than complete cases.

This study has several limitations. First, this was a single-center, retrospective study of a predominantly male Chinese cohort with interstitial lung disease and chronic obstructive pulmonary disease as the leading indications, which may limit the generalizability of these trajectories to Western or multi-center populations. Nonetheless, the cohort’s composition was typical of lung transplant populations. Second, the Ishii score is a bedside screen rather than a reference standard, and it incorporates age as one of its components. We chose it because the study required repeated assessment that imaging cannot practically provide. Accordingly, these trajectories represent longitudinal patterns of Ishii-defined sarcopenia risk rather than progression of sarcopenia confirmed by consensus diagnostic criteria. In the age-component-removal sensitivity analysis, individual class assignments agreed with the primary analysis for 94.7% of recipients. Third, the analysis did not include several muscle-modifying variables, such as daily physical activity, cumulative corticosteroid exposure, post-transplant infection, rehabilitation adherence, and protein intake, because these were not routinely recorded in the retrospective data, so residual confounding cannot be excluded. These unmeasured postoperative factors could either support or hinder muscle recovery and thereby influence trajectory shape and class membership. The associated factors are therefore best regarded as markers for risk stratification rather than causal influences. Fourth, loss of the 12-month assessment was more frequent among recipients in the high-risk trajectory (23.3%) than among those in the low-risk trajectory (11.2%), so recovery in the high-risk trajectory may be estimated optimistically. The analysis using inverse probability weighting yielded class proportions similar to those of the primary analysis. Both analyses rely on the assumption that data are missing at random. Residual bias cannot be excluded.

### Future directions

Verification of these trajectories with reference-standard measures of muscle mass in larger, multi-center cohorts will be an important next step for translating this stratification into clinical practice. Relating trajectory class to longer-term clinical outcomes would further establish its prognostic value. Interventional trials are needed to evaluate the value of trajectory-based nutrition and exercise programs in high-risk recipients.

## Conclusion

Growth mixture modeling identified three distinct Ishii score trajectories during the first year after lung transplantation, with recovery varying markedly between recipients. This heterogeneity calls for a trajectory-based approach to postoperative sarcopenia. Older age, lower preoperative BMI and FEV1%pred, longer ICU stay, and higher nutritional risk distinguished the high-risk trajectory, although causality cannot be confirmed. Measured repeatedly at the bedside, the Ishii score offers a practical way to detect high-risk recipients early and to guide their care from before transplantation onward.

## Data Availability

The datasets presented in this article are not readily available because they contain clinical information from lung transplant recipients and are subject to institutional restrictions on data sharing. Requests to access the datasets should be directed to the corresponding author, Fei Zeng (zengfei@zju.edu.cn).
