# Supplementary material for: Sarcopenia trajectories and associated factors after lung transplantation: a growth mixture model study
Source: Front Med (Lausanne). 2026 Sep 1;13:1920710. doi: 10.3389/fmed.2026.1920710 (PMC13574608; doi:10.3389/fmed.2026.1920710)
Supplement: Supplementary file 1 [file Table_1.docx]

**Supplementary Material**

**Supplementary Table S1.** Missing-data diagnostics for the Ishii assessments

*Panel A. Assessments available and missing at each time point*

| **Time point** | **Assessed, n** | **Missing, n (%)** |
| --- | --- | --- |
| Preoperative | 264 | 0 (0.0) |
| 1 week | 251 | 13 (4.9) |
| 1 month | 253 | 11 (4.2) |
| 3 months | 240 | 24 (9.1) |
| 6 months | 228 | 36 (13.6) |
| 12 months | 225 | 39 (14.8) |

*Panel B. Baseline and perioperative characteristics of recipients with complete versus incomplete assessments*

| **Characteristic** | **Complete (n = 205)** | **Incomplete (n = 59)** | ***p*** |
| --- | --- | --- | --- |
| Age, years | 59.0 (52.0, 65.0) | 57.0 (50.5, 64.5) | 0.236 |
| Male, n (%) | 181 (88.3) | 46 (78.0) | 0.072 |
| BMI, kg/m² | 20.9 (18.0, 23.7) | 19.3 (16.6, 21.9) | 0.055 |
| FEV1%pred | 46.3 (30.3, 57.3) | 42.2 (26.5, 61.9) | 0.988 |
| ICU stay, days | 4.0 (3.0, 7.0) | 5.0 (3.0, 8.0) | 0.401 |
| NRS 2002 | 2.0 (1.0, 4.0) | 2.0 (1.0, 4.0) | 0.982 |
| Preoperative Ishii | 115.1 (89.4, 137.8) | 120.4 (93.6, 152.6) | 0.217 |

*Panel C. Logistic regression of loss of the 12-month assessment on baseline and perioperative characteristics*

| **Predictor** | **OR (95% CI)** | ***p*** |
| --- | --- | --- |
| Preoperative BMI | 0.87 (0.77–0.98) | 0.024 |
| Preoperative FEV1%pred | 1.00 (0.97–1.02) | 0.640 |
| ICU stay | 1.08 (1.03–1.13) | 0.001 |
| NRS 2002 score | 0.72 (0.54–0.96) | 0.026 |

*Complete denotes recipients assessed at all six time points and incomplete those missing one or more. In Panel B, continuous variables are median (P25, P75) compared with the Mann-Whitney U test and categorical variables are n (%) compared with the χ² test. Panel C is a multivariable logistic regression of loss of the 12-month Ishii assessment on baseline and perioperative characteristics, restricted to recipients with complete covariate data (n = 247, 35 losses). OR, odds ratio; CI, confidence interval; BMI, body mass index; FEV1%pred, percent-predicted forced expiratory volume in one second; ICU, intensive care unit; NRS 2002, Nutritional Risk Screening 2002.*

**Supplementary Table S2.** Ishii scores at six time points among sarcopenia trajectory classes (mean and 95% CI, points)

| **Class** | **n** | **Preoperative** | **1 week** | **1 month** | **3 months** | **6 months** | **12 months** |
| --- | --- | --- | --- | --- | --- | --- | --- |
| High-risk | 60 | 156.0 (153.8–158.1) | 161.9 (159.3–164.6) | 164.2 (161.1–167.3) | 159.2 (156.2–162.2) | 154.3 (150.7–157.8) | 149.6 (146.3–152.9) |
| Moderate-risk | 97 | 123.2 (120.3–126.1) | 140.1 (138.1–142.1) | 138.1 (135.3–141.0) | 122.2 (119.5–124.9) | 106.2 (103.3–109.1) | 94.6 (91.7–97.6) |
| Low-risk | 107 | 88.7 (86.6–90.7) | 123.4 (121.1–125.6) | 102.7 (99.9–105.4) | 79.0 (76.6–81.5) | 74.4 (71.5–77.4) | 65.7 (63.2–68.1) |
| Overall | 264 | 116.7 (113.2–120.1) | 138.3 (136.0–140.5) | 129.4 (125.9–132.8) | 111.7 (107.5–116.0) | 102.8 (98.6–107.0) | 93.6 (89.3–98.0) |

*n denotes the number of recipients assigned to each trajectory. Means and 95% CIs were calculated using the observations available at each time point. CI, confidence interval.*

**Supplementary Table S3.** Robustness of the three-class trajectory solution across sensitivity models

| **Model** | **n** | **Class proportion (%)** | **Entropy** |
| --- | --- | --- | --- |
| Primary (FIML) | 264 | 22.7 / 36.7 / 40.5 | 0.906 |
| Edema-adjusted GMM | 264 | 22.3 / 37.1 / 40.5 | 0.925 |
| Age-component-removed score GMM | 264 | 22.3 / 33.7 / 43.9 | 0.906 |
| IPW-weighted GMM | 225 | 22.6 / 37.0 / 40.4 | 0.940 |

*Class proportions are shown as high-risk / moderate-risk / low-risk. The edema-adjusted model recomputed Ishii scores after correcting calf circumference for edema, and its class assignment was concordant with the primary model in 98.9% of recipients. The age-component-removed model used the sum of the sex-specific grip-strength and calf-circumference component scores. Under the same three-class model specification, class-assignment agreement with the primary model was 94.7%. The IPW-weighted model reweighted completers by the inverse probability of retention to address differential attrition. Class proportions were similar across all four models. FIML, full-information maximum likelihood; IPW, inverse-probability weighting; GMM, growth mixture model.*

**Supplementary Table S4.** Physical performance at 12 months by sarcopenia trajectory class

| **Variable** | **High-risk (n = 46)** | **Moderate-risk (n = 84)** | **Low-risk (n = 95)** | ***p*** |
| --- | --- | --- | --- | --- |
| SPPB, median (P25, P75) | 8 (6, 10) | 10 (8, 12) | 11 (9, 12) | <0.001 |
| Functional limitation, n (%) | 33 (71.7%) | 40 (47.6%) | 27 (28.4%) | <0.001 |

*Post-hoc exploratory analysis. n denotes recipients with a 12-month SPPB assessment. Functional limitation was defined as an SPPB score of 9 or below. p values from the Kruskal-Wallis test (SPPB) and the χ² test (functional limitation). SPPB, Short Physical Performance Battery; P25, 25th percentile; P75, 75th percentile.*

**Supplementary Table S5.** Sensitivity analyses: multinomial logistic regression of sarcopenia trajectory classes, comparing the primary and subset analyses (odds ratio [95% CI])

| **Variable** | **Primary (multiple imputation, n = 264)** | **Edema-excluded (n = 157)** | **Complete-case (n = 188)** |
| --- | --- | --- | --- |
| ***High-risk vs low-risk (reference)*** |  |  |  |
| Age (years) | 1.084 (1.036–1.134)* | 1.111 (1.034–1.193)* | 1.060 (1.005–1.117)* |
| Preoperative BMI | 0.810 (0.705–0.931)* | 0.766 (0.634–0.925)* | 0.821 (0.691–0.975)* |
| **Primary diagnosis** |  |  |  |
| Interstitial lung disease | – | – | – |
| COPD | 1.141 (0.382–3.412) | 1.265 (0.321–4.996) | 1.285 (0.379–4.357) |
| Other | 0.596 (0.180–1.973) | 0.629 (0.129–3.067) | 0.441 (0.106–1.835) |
| Preoperative FEV1%pred | 0.974 (0.948–1.000)* | 0.963 (0.932–0.994)* | 0.984 (0.955–1.014) |
| NRS 2002 score | 1.392 (1.055–1.836)* | 1.184 (0.821–1.706) | 1.369 (0.981–1.910) |
| ICU stay | 1.109 (1.026–1.198)* | 1.087 (0.988–1.195) | 1.067 (0.955–1.193) |
| Postoperative stay | 1.020 (0.993–1.047) | 1.039 (1.000–1.080) | 1.028 (0.995–1.061) |
| ***Moderate-risk vs low-risk (reference)*** |  |  |  |
| Age (years) | 1.042 (1.010–1.076)* | 1.032 (0.988–1.077) | 1.028 (0.992–1.065) |
| Preoperative BMI | 0.928 (0.845–1.019) | 0.905 (0.794–1.032) | 0.934 (0.834–1.046) |
| **Primary diagnosis** |  |  |  |
| Interstitial lung disease | – | – | – |
| COPD | 0.650 (0.260–1.621) | 0.888 (0.267–2.955) | 0.655 (0.237–1.815) |
| Other | 0.879 (0.391–1.977) | 0.612 (0.202–1.856) | 0.665 (0.256–1.729) |
| Preoperative FEV1%pred | 0.988 (0.970–1.007) | 0.998 (0.973–1.024) | 0.994 (0.972–1.016) |
| NRS 2002 score | 1.298 (1.046–1.611)* | 1.241 (0.909–1.696) | 1.294 (1.004–1.669)* |
| ICU stay | 1.070 (0.994–1.151) | 1.067 (0.975–1.168) | 1.082 (0.981–1.192) |
| Postoperative stay | 1.005 (0.982–1.029) | 1.021 (0.985–1.058) | 1.004 (0.977–1.032) |

*Asterisk, p < 0.05. The primary analysis used multiple imputation (n = 264); the edema-excluded (n = 157) and complete-case (n = 188) regression analyses were restricted to recipients with complete covariate data. For primary diagnosis, interstitial lung disease is the reference (–). OR, odds ratio; CI, confidence interval; BMI, body mass index; FEV1%pred, percent-predicted forced expiratory volume in one second; ICU, intensive care unit; NRS 2002, Nutritional Risk Screening 2002.*
